# Highly efficient neuronal gene knockout in vivo by CRISPR-Cas9 via neonatal intracerebroventricular injection of AAV in mice

**DOI:** 10.1038/s41434-021-00224-2

**Published:** 2021-02-08

**Authors:** Sam Hana, Michael Peterson, Helen McLaughlin, Eric Marshall, Attila J. Fabian, Olivia McKissick, Kathryn Koszka, Galina Marsh, Michael Craft, Shanqin Xu, Alexander Sorets, Tess Torregrosa, Chao Sun, Chris E. Henderson, Shih-Ching Lo

**Affiliations:** Biogen Inc., Cambridge, MA USA

**Keywords:** Cellular neuroscience, Molecular biology

## Abstract

CRISPR-Cas systems have emerged as a powerful tool to generate genetic models for studying normal and diseased central nervous system (CNS). Targeted gene disruption at specific loci has been demonstrated successfully in non-dividing neurons. Despite its simplicity, high specificity and low cost, the efficiency of CRISPR-mediated knockout in vivo can be substantially impacted by many parameters. Here, we used CRISPR-Cas9 to disrupt the neuronal-specific gene, NeuN, and optimized key parameters to achieve effective gene knockout broadly in the CNS in postnatal mice. Three cell lines and two primary neuron cultures were used to validate the disruption of NeuN by single-guide RNAs (sgRNA) harboring distinct spacers and scaffold sequences. This triage identified an optimal sgRNA design with the highest NeuN disruption in in vitro and in vivo systems. To enhance CRISPR efficiency, AAV-PHP.B, a vector with superior neuronal transduction, was used to deliver this sgRNA in Cas9 mice via neonatal intracerebroventricular (ICV) injection. This approach resulted in 99.4% biallelic indels rate in the transduced cells, leading to greater than 70% reduction of total NeuN proteins in the cortex, hippocampus and spinal cord. This work contributes to the optimization of CRISPR-mediated knockout and will be beneficial for fundamental and preclinical research.

## Introduction

Over the past century, genetic studies in invertebrate systems, such as bacteria, yeast, *C. elegans* and *Drosophila* have provided profound insights into our fundamental understanding of metazoan biology and disease. However, traditional genetic analyses in vertebrate models have been limited for numerous reasons, including high costs, laboratory space restrictions, and time-consuming mutant germline engineering procedures [1]. Recent advances in gene editing technologies, from zinc-finger nucleases to TALEN and CRISPR, provide the ability to manipulate genomes at targeted loci with high specificity. Due to its ease of implementation, the CRISPR-Cas endonuclease system is the most widely employed and has become a powerful gene editing tool in basic and translational research as well as therapeutic development [2–5].

Among its many applications, targeted gene disruption by the CRISPR-Cas9 endonuclease system has enabled the rapid generation of mammalian models by gene knockout mutations [6]. In this scheme, the Cas9 enzyme, bound to specificity-determining guide RNA, is targeted to a genomic loci and introduces a double-strand break in the genomic DNA. These breaks can be repaired via non-homologous end-joining (NHEJ) unless a DNA repair template is present for homology-directed repair. NHEJ introduces insertions and deletions (indels) and can lead to frameshift mutations and premature a stop codon resulting in gene disruption. Gene knockouts occurs when both alleles of a gene are disrupted by the CRISPR-Cas9 system.

The prototypical CRISPR-Cas9 system contains spCas9, an RNA-guided DNA endonuclease, and an RNA duplex comprised of a CRISPR RNA (crRNA) repeats and a transactivating crRNA (tracrRNA). In 2012, Jinek et al. first combined the native crRNA:tracrRNA duplex into a chimeric, single-guided RNA (sgRNA), connected by a linker loop, to simplify CRISPR-Cas implementation [7]. The 20-nt “spacer” sequence within the sgRNA can be altered to direct spCas9 towards a target locus of interest flanked by a protospacer adjacent motif (PAM). The architectures of the scaffold portion of an sgRNA, formed by the crRNA repeats and tracrRNA secondary structure, were subsequently modulated to enhance stability or activity. To date, the most commonly used sgRNA scaffold for spCas9 is an extension from the original scaffold, allowing for more efficient Cas9 loading [8, 9]. Recently, more modifications to the sgRNA scaffold have been made to further improve CRISPR efficiency, including lengthening of the hairpin secondary structure and the incorporation of biochemical changes to the sugar-phosphate backbone [10–14].

Efficient delivery of CRISPR-Cas9 to the CNS is key to robust neuronal gene disruption in vivo. Adeno-associated viruses (AAVs) are non-pathogenic, safe and capable of transducing both dividing and non-diving cells, and thus present a unique opportunity of accessing neurons in the CNS for CRISPR knockout [15, 16]. Previously, the CRISPR-Cas9 system has been used to locally knockout NeuN, a gene specifically expressed in neurons, in adult mouse brain [10, 17]. Stereotactic injection of AAV1/2 carrying NeuN-targeting sgRNA (sgNeuN) resulted in robust reduction of NeuN expression in the vicinity of the injection site in the cortex [10]. However, global knockout of a neuronal gene in the CNS remained a challenge. Compared to many natural-occurring AAV serotypes, neurotropic AAV9 enables gene transfer across the blood brain barrier and leads to widespread cargo expression throughout the CNS [18]. AAV-PHP.B, an engineered AAV9 variant, was shown to broadly transduce CNS tissues at very high efficiencies, upon systemic delivery, much improved from AAV9 [18–22]. Furthermore, three studies showed successful delivery of CRISPR-Cas9 by AAV in the eye, inner ear and the heart for gene editing in mice [23–25]. The utility of AAV-PHP.B in aiding neuronal gene editing by CRISPR-Cas9 in the brain and the spinal cord is yet to be explored.

In the present study, we sought to optimize the approach for extensive NeuN gene knockout by CRISPR-Cas9 throughout the mouse CNS. We first established in vitro methods to select for a highly efficient sgRNA and then we incorporated key technical elements to enhance CNS delivery of this sgRNA in Cas9 knock-in mice. Three NeuN spacer sequences were selected and were each paired with two different sgRNA scaffolds for comparison of CRISPR activity. We showed that one combination of spacer sequence and sgRNA scaffold consistently resulted in marked depletion of NeuN proteins in multiple in vitro systems, and when delivered in mice, also showed greater NeuN reduction than the other sgRNAs tested. Importantly, highly efficient CRISPR-mediated NeuN disruption in vivo was achieved by adopting AAV-PHP.B to deliver this sgRNA via neonatal intracerebroventricular (ICV) injection directly into the cerebral spinal fluid (CSF) [15, 26]. Examination of NeuN protein expression via immunohistochemistry confirmed robust NeuN knockout in multiple brain and spinal cord regions. Indel analysis by next-generation sequencing (NGS) revealed an overwhelming majority of the transduced cells in the cortex contained biallelic gene edits with frameshift mutations at the targeted NeuN locus. These results demonstrate the utility of our approach to facilitate the generation of knockout mutations in neuronal genes in vivo and open the possibility for rapid assessment of their normal functions as well as their role in the pathology and etiology of CNS diseases.

## Materials and methods

### Animals

H11-Cas9 mice on B6;129 background [Igs2tm1.1(CAG-cas9*)Mmw/J; laboratory of M. Winslow, Stanford University, Stanford, CA] (Stock #:027650) constitutively expressing codon-optimized *Streptococcus pyogenes* Cas9 (spCas9) were obtained from the Jackson Laboratory [27]. Homozygous H11-Cas9 mice were crossed to generate animals used in all experiments of this study. Mice were housed in a 12/12 h light/dark cycle in a temperature-controlled room (22–24 °C) with access to food pellet and water provided *ad libitum*. Experimenters were blind to the treatments. All animal use and treatments were approved by the Institutional Animal Care and Use Committee and the National Institute of Health Guide for the Care and Use of Laboratory Animals.

### Single-guide RNA (sgRNA) design, plasmid construction

The NeuN-targeting spacer sequences #1–3 were selected based on on-target and off-target score provided by Benchling (https://benchling.com/) (San Francisco, CA, USA) bioinformatic output against the mouse NeuN gene [28, 29]. Each spacer sequence was constructed in two sgRNA scaffolds developed by Feng Zhang’s lab (termed FZ) and Bo Huang’s lab (termed BH) respectively for improved binding of spCas9 (Table S2) [10, 30]. Each construct contains a U6 promoter that drives the expression of an sgRNA and a CAG promoter that drives the expression of a reporter gene, mCherry, turboGFP (termed tGFP) or eGFP (Table S1). The WPRE regulatory element and hGHpA polyadenylation signal were also included in the 3′ end of the constructs (Table S1). The reporter eGFP was fused to the KASH domain to facilitate nuclei isolation for indel analysis. For controls, either sgRNA was omitted from the constructs (Control) or an sgRNA targeting the LacZ (b-galactosidase) gene (termed sgLacZ) was used (Table S2). All plasmids were verified by Sanger sequencing.

### AAV vector production and purification

AAV vector production and purification were performed by Virovek (Hayward, California, USA) as previously described [31]. Briefly, Sf9 insect cells were cultured at 28 °C to ~107 cells/mL in Sf-900 II serum-free medium containing 100 U/mL of penicillin and 100 μg/mL streptomycin. The Sf9 cells were diluted to ~5 × 106 cells/mL before infection. Triple infection with baculovirus vector coding for the replication proteins (Bac-inRep), the structural proteins (Bac-inCap) and AAV vector genomes (BacITR) was carried at 28 °C for 3 days to produce AAV9 and AAV-PHP.B vectors (Table S1). After 3 days of infection, the cell pellets were centrifuged at 3,000 rpm for 15 mins. The cell pellets were lysed, and the released nucleic acids were digested. The cell lysates were cleared by centrifugation at 8,000 rpm for 30 mins. The lysates were then loaded onto a SW28 centrifuge tube containing a discontinuous CsCl gradient (5 mL of 1.55 g/cc and 10 mL of 1.32 g/cc) for centrifugation at 28,000 rpm for 16 h at 15 °C. The rAAV fraction was collected by puncturing the centrifuge tube with a syringe needle and the rAAV was subjected to another round of CsCl ultracentrifugation. The rAAV was collected by a syringe needle and the rAAVs were desalted into phosphate-buffered saline with a PD-10 desalting column (GE HealthCare, Piscataway, NJ). AAV9 and AAV-PHP.B titers were determined by qRT-PCR. Virovek (Hayward, California, USA).

### Cell culture and transfection

COS1 cells (ATCC, Manassas, VA) were maintained in Dulbecco’s Modified Eagle’s Medium (DMEM) supplemented with 10% heat-inactivated fetal bovine serum (HI-FBS), 1% Penicillin–Streptomycin (Pen-Strep) and 2 mM l-glutamine—complete medium—and incubated at 37 °C and 5% CO_2_. FUGENE HD (Promega, San Luis Obispo, CA, USA) was used for transient transfection according to the manufacturer’s instructions. Briefly, pCMV6-Mm.NeuN-tGFP (OriGene, Rockville, MD), pVAX1-FLAG-spCas9 (a gift from Douglas Larigan) and pFB-U6-sgNeuN-CAG-mCherry expression plasmids were mixed in a 1:4:5 ratio, and incubated with FUGENE HD in Opti-MEM media (Thermo Fisher Scientific, Waltham, MA, USA). The mixture was added to COS1 cells, in complete medium without Pen-Strap, at 80% confluency. A replicate of two were tested per sgNeuN treatment for all in vitro assays. Cell lysates were collected in Novex Tris-Glycine SDS Sample Buffer (Thermofisher, LC2676) 24 h post transfection.

HeLa cells stably expressing spCas9 (GeneCopoeia, Rockville, MD, USA) were maintained in DMEM supplemented with 10% HI-FBS, 2 mM l-glutamine and hygromycin (250 µg/mL) and incubated at 37 °C and 5% CO_2_. Cells were co-transfected with pCMV6-Mm.NeuN-tGFP and pFB-U6-sgNeuN-CAG-mCherry-WPRE-hGHpA expression plasmids at 1:9 ratio, using FUGENE HD and were lysed 24 h after transfection.

The Neuro-2a cells with tetracycline-inducible spCas9 expression (GeneCopoeia) were maintained in DMEM supplemented with 10% HI-FBS, 2 mM l-glutamine and hygromycin (100 µg/mL final)—complete medium—and incubated at 37 °C and 5% CO_2_. Neuro-2a cells were seeded at a density to ensure ~80% confluency on the day of transfection. On the next day, cells were treated with various concentrations of the doxycycline to induce spCas9 expression (0.01, 0.1, and 1 µg/mL). After 24 h, Neuro-2a cells were co-transfected with pCMV6-Mm NeuN-tGFP and pFB-U6-sgNeuN-CAG-mCherry-WPRE-hGHpA at 1:9 ratio, using FUGENE HD, and were lysed 24 h after transfection.

Mouse primary hippocampal and cortical neuron cultures were prepared from H11-Cas9 embryos on embryonic day 16 (E16) as previously described [32]. Primary neurons were transduced with AAV9-U6-sgRNA-CAG-mCherry-WPRE-hGHpA (250 K MOI) at days in vitro (DIV) 3 and cells were lysed at DIV15 for immunoblotting analysis of endogenous NeuN proteins.

### Western blotting and antibodies

All cell lysates were heated at 70 °C to denature proteins, sonicated to shear genomic DNA and then electrophoresed through Tris-Glycine gels or NuPAGE Bis-Tris gels (Thermo Fisher Scientific) for SDS-PAGE separation by mass. Proteins were transferred to nitrocellulose membranes using the Trans-Blot Turbo Transfer System (Bio-Rad, Hercules, CA, USA). The blocking of membranes and subsequent antibody incubations were performed using Odyssey Blocking Buffer according to the manufacturer’s instructions (LI-COR Biosciences, Lincolin, NE, USA). Primary antibodies against mouse NeuN (12943, Cell Signaling Technologies, Danvers, MA, USA; ab177487, Abcam), β-Tubulin (926–42211, LI-COR Biosciences), β-actin (926–42210, LI-COR Biosciences), FLAG (F1804, Millipore Sigma, Burlington, MA), mCherry (ab167453, Abcam, Cambridge, United Kingdom), GAPDH (ab8245, Abcam) and turboGFP (TA150041-100, OriGene) were obtained from commercial sources. LI-COR’s secondary antibodies, IRDye 800CW-conjugated and IRDye 680-conjugated antibodies, were used based on the manufacture’s protocol. Immunoblot signals were visualized by LI-COR’s Odyssey CLx infrared imaging system and quantified by Image Studio Lite (LI-COR Biosciences).

### Neonatal intracerebroventricular (ICV) injection of AAV

On postnatal day 0 (P0), neonatal mice were anesthetized by hypothermia for 2–4 min until movement ceased. Cryo-anesthetized pups were injected with an AAV solution diluted in phosphate-buffered saline containing FastGreen dye (final concentration: 0.25%) into the lateral ventricle(s). For Fig. 3, 2 μL of AAV9 at a total dose of 4.3 × 10^10^ vector genome copies (GC) were administered in each pup; for Fig. 4, 1 × 10^11^ GC of AAV-PHP.B in 2 μL and 2 × 10^11^ in 4 μL were administered for the low dose group and the high dose group, respectively; for Figs. 5–7, 2 × 10^11^ GC of AAV-PHP.B in 4 μL were administered. Pups within each litter were randomized for the treatments. Injection sites were located 1 mm lateral to the superior sagittal sinus, halfway between lambda and bregma, to a depth of 2 mm [33]. Injections were performed with a 33-gauge, 10 µL, 45° bevel Hamilton syringe (Hamilton Company, Reno, NV, USA) inserted perpendicular to the surface of the skull [33]. Injection efficiency was monitored by the spread of the dye throughout the lateral and the third ventricles. Mice were weaned at 4 weeks of age and sacrificed at 6 weeks of age for western blotting and immunohistochemistry analyses, or at 5 weeks of age for indel analysis. Experimenters were blind to the treatments.

### Immunohistochemistry and image analysis

Sagittal brain hemispheres were fixed in 10% neutral buffered formalin for 48–72 h and the spinal cord was fixed for 24–48 h. The brain was processed and embedded midline-down using Surgipath Paraplast Plus (Leica Biosystems, Wetzlar, Germany) paraffin blocks. The spinal cords were bisected and embedded vertically. Double immunofluorescence was performed on a Ventana DISCOVERY ULTRA (Roche Diagnostics, Risch-Rotkreuz, Switzerland) with anti-mCherry, anti-NeuN antibodies and DAPI sequentially according to the manufacturer’s protocols. Briefly, 5 µm thick sections were deparaffinized and rehydrated. The sections were subjected to antigen retrieval using ULTRA Cell Conditioning Solution (Roche Diagnostics) followed by incubation with first primary rabbit polyclonal anti-mCherry antibody (0.025 μg/mL, Abcam, ab167453) for 32 min and then incubated with polymer-based secondary antibody-Discovery OmniMap anti-Rb HRP and DISCOVERY Rhodamine Kit (Roche Diagnostics) for detection. The anti-mCherry antibody and bound HRP conjugates were denatured and neutralized, respectively, the sections were then incubated with a primary rabbit monoclonal anti-NeuN (0.0325 µg/mL, Cell Signaling Technology, 24307) for 32 min. The signal was detected with polymer-based secondary antibody-Discovery OmniMap anti-Ms HRP and DISCOVERY Cy5 Kit (Roche Diagnostics). Whole slides were scanned at ×20 or ×40 magnification at identical exposure times on a slide scanner using DAPI, TRITC and Cy5 filters. Custom analysis algorithms were made with Visiopharm software (Horsholm, Denmark). Images were manually annotated and analyzed by a blinded analyst. In each region, cells were individually identified by the presence of DAPI and binned as either positive or negative for mCherry and NeuN by meeting a mean signal intensity threshold for rhodamine and Cy5, respectively. Prior to binning, Cy5 signal bleed through into the rhodamine channel was measured and spectrally removed at the pixel level. Formulas to calculate neuronal presence in cells, percent transduction and percent CRISPR efficiency are provided (Table S3).

### Purification of nuclei, FACS, and NGS sequencing

Five weeks post injection of AAV-U6-sgRNA-CAG-eGFP-KASH-WPRE-hGHpA, cortices were dissected, snap frozen and stored at −80 °C. Frozen cortices were thawed and homogenized in 1.5 mL ice-cold homogenization buffer (HBSS, 25 mM HEPES). The homogenate was passed through a 250 µm filter and spun at 600 × g for 5 min at 4 °C. The cell pellet was gently resuspended in 1 mL FBS and subsequently in 9 mL of 33% Percoll solution (GE Healthcare, Chicago, IL, USA) containing HBSS and 16.7 mM HEPES. An additional 1 mL of 10% FBS solution containing HBSS and 22.5 mM HEPES was carefully layered on the top of cell suspension layer containing 30% Percoll. Density gradient centrifugation was performed at 800 × g for 15 min at 4 °C (1 acceleration and 1 brake). The supernatant was removed, and the nuclei pellet was resuspended and washed in FACS buffer (HBSS, 1% BSA, 2 mM EDTA, 25 mM HEPES, 0.09% sodium azide). 5 × 10^5^ intact eGFP positive nuclei labeled with Vybrant DyeCycle Violet Stain (Thermo Fisher Scientific) were isolated by FACS using MoFlo Astrios EQ (Beckman Coulter, Brea, CA, USA). For Fig. 7a, DNA was isolated from the pooled nuclei by DNeasy Blood & Tissue Kit (catalog #: 69504) according to the manufacturer’s instruction (Qiagen, Hilden, Germany).

Primers (Table S2) with Illumina adaptors were used to amplify a region of the murine NeuN surrounding the sgRNA-targeted locus, using KAPA HiFi HotStart ReadyMix (Kapa Biosystems, Wilmington, MA, USA). The library was constructed by indexing individual samples with Illumina’s Nextera XT indices (Illumina, San Diego, CA, USA) with limited PCR cycles. The library was pooled by volume and purified using AMPure XP beads (Beckman Coulter). The final library pool was quantified by KAPA Library Quantification Kit (KAPA Biosystems) and loaded on to Illumina’s MiSeq at 10 pM for 2 × 150 bp cycle run.

### Statistics

All data are expressed as means ± SD. One-way ANOVA followed by Dunnett’s or Tuckey’s multiple comparison tests were performed to determine the significance of differences between mean values, and the null hypothesis was rejected at *P* < 0.05. GraphPad Prism 8 (San Diego, CA, USA) was used to graph all data and to compute statistical analysis.

## Results

### Multiple in vitro assays identify a potent NeuN-targeting sgRNA for subsequent use in vivo to achieve reliable NeuN protein reduction

**Fig. 1:**
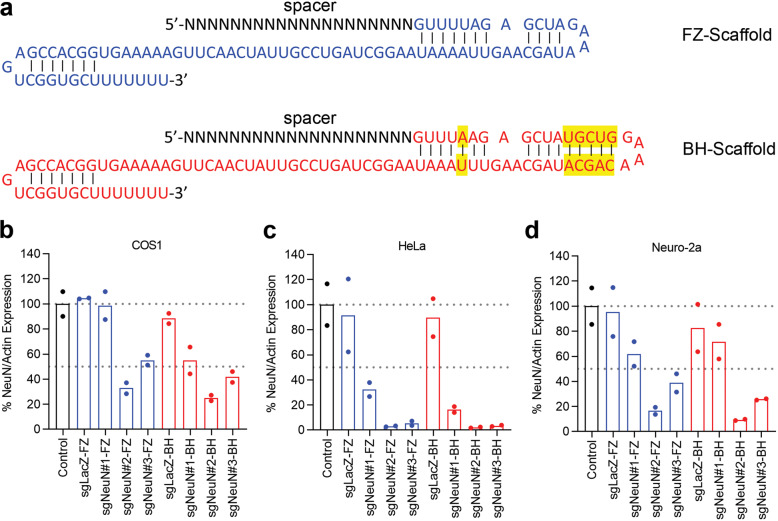
Cell line assays of sgNeuN#1–3 and the effect of sgRNA scaffolds, FZ and BH, on NeuN protein. **a** The sequences of the sgRNA scaffolds with differences highlighted in yellow. sgNeuN#2 reduces NeuN protein in **b** COS1 cells, **c** HeLa cells, and **d** Neuro-2a cells. In all immortalized cells, the BH scaffold is slightly more efficient than the FZ scaffold. COS1 cells were triple transfected with plasmids carrying mouse NeuN, FLAG Cas9 and sgRNAs. HeLa cells, stably expressing Cas9, were co-transfected with mouse NeuN gene and sgRNAs. Neuro-2A cells with doxycycline inducible Cas9 were co-transfected with mouse NeuN and sgRNAs. NeuN expression was first normalized to actin and then to Control, construct not expressing sgRNA. Data represent means of *n* = 2 technical replicates. Refer to Fig. S1–S3 for representative gel images. Scaffold sgRNA sequences: FZ-Feng Zheng [Blue] and BH-Bo Huang [Red]. (color figure online).

NeuN (Rbfox3) is a commonly used gene marker for mature neurons, and almost all neurons express NeuN, except for cerebellar Purkinje neurons, inferior olive neurons and mitral cells in the olfactory bulb [17, 34]. The NeuN gene was selected for CRISPR-mediated disruption in this study to assess CRISPR efficiency in neurons, as the NeuN knockout mice develop normally without gross abnormality at young adult age [35]. In addition, NeuN is localized in the nucleus and thereby allows easy quantification of neuronal cell body via immunohistochemistry [34]. Three spacers targeting the mouse NeuN gene (termed sgNeuN) were selected based on high on-target scores and low off-target scores predicted by Benchling’s algorithm [28, 29]. To construct the full sgRNAs, each of the three spacers was paired with one of the two sgRNA scaffolds, yielding six different combinations for sgRNA design. The first sgRNA scaffold tested (referred to as FZ in this article) was reported by Hsu et al. [8, 10] (Fig. 1a). The second sgRNA scaffold tested (referred to as BH in this article) was reported by Chen et al. and is noted for its improved Cas9 binding affinity [14] (Fig. 1a). To determine which sgRNAs can robustly disrupt exogenous expression of NeuN regardless of the cellular milieu, in vitro assays were performed in three different cell lines: COS1, HeLa and Neuro-2a cells. Cells were co-transfected with overexpression vectors for Cas9, the mouse NeuN gene, and sgNeuN-FZ or sgNeuN-BH, lysed at 24 h (COS1 and HeLa) or 48 h (Neuro-2a) and analyzed for NeuN protein levels via western blotting. Spacer sequences #2 and #3 consistently decreased NeuN protein levels in all cell types with varying potencies (Fig. 1b–d). In COS1 cells, sgNeuN#2-BH resulted in ~75% reduction of NeuN expression, outperforming sgNeuN#3-BH which resulted in ~59% reduction (Fig. 1b). In Neuro-2a cells, ~84% of NeuN protein was reduced when treated with sgNeuN#2-FZ compared to ~62% of sgNeu#3-FZ (Fig. 1d). In HeLa cells, sgNeuN#2 and sgNeuN#3 for both the BH and FZ scaffolds achieved >95% reduction in NeuN protein (Fig. 1c). In addition, when paired with the poorly efficient spacer sequence #1, the BH scaffold improved the potency of sgNeuN#1, leading to ~45% NeuN reduction in COS1 cells (Fig. 1b). Overall, this data demonstrates that sgNeu#2 and sgNeu#3 were capable of lowering NeuN protein expression in all cell types. Between the two, spacer sequence #2 consistently performed better than spacer #3 (Fig. 1b, d).

**Fig. 2:**
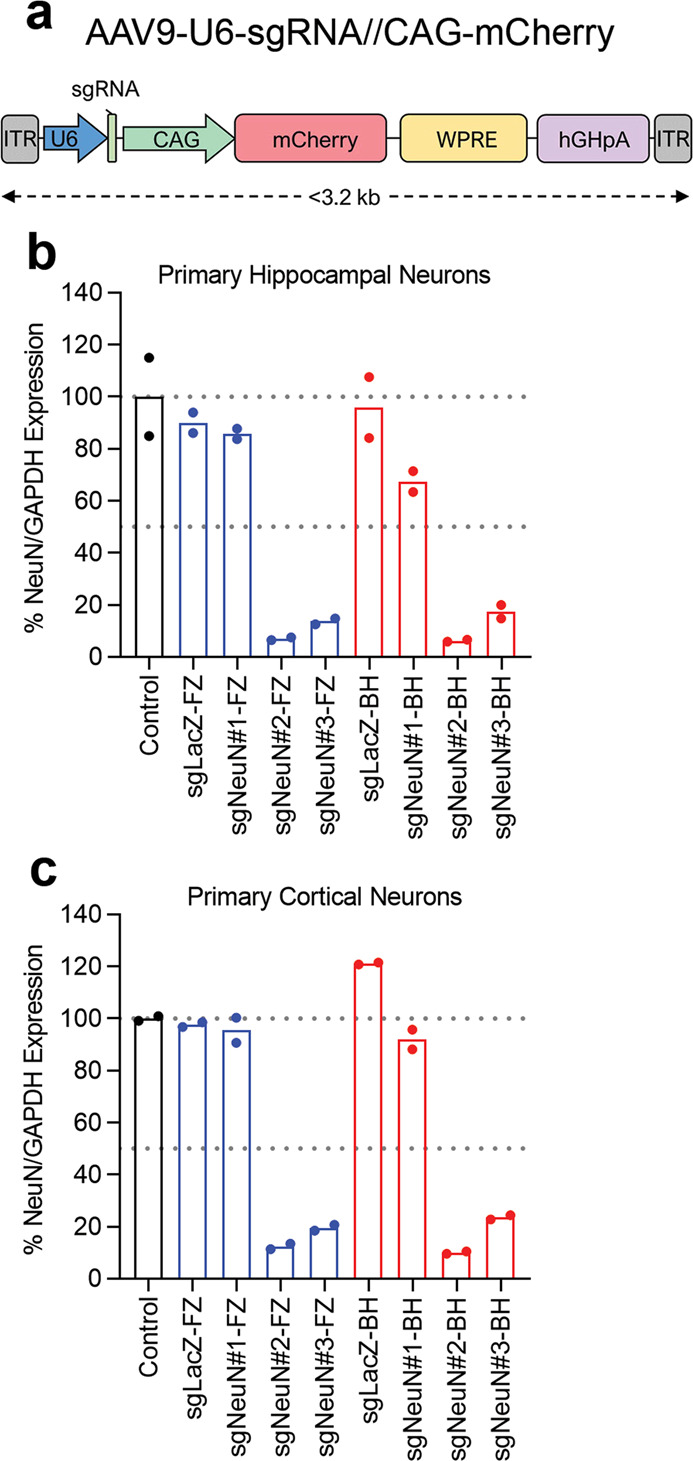
Primary neuronal screen of sgNeuN#1–3 and two sgRNA scaffolds. **a** Vector map of the AAV9 transgene. Spacers #2 and #3 achieves robust reduction in **b** primary hippocampal neurons and **c** primary cortical neurons. Embryonic stage 16 primary neurons were transduced with 250 K AAV9 carrying sgRNAs constructs. Endogenous NeuN expression was normalized to GAPDH and then to Control, construct not expressing any sgRNA. Data represent means of *n* = 2 technical replicates. Refer to Fig. S4–S5 for representative gel images. FZ-Feng Zheng [Blue] and BH-Bo Huang [Red]. (color figure online).

Next, we validated the potency of sgRNA spacers and scaffolds in decreasing the expression of endogenous NeuN proteins in primary mouse neuron cultures prepared from E16 Cas9 embryos. The sgRNA constructs were packaged in AAV9 for in vitro transduction (Fig. 2a). Hippocampal and cortical neuron cultures were transduced with viral particles at 250 K MOI (multiplicity of infection) on DIV3 and lysed on DIV13 or DIV15 for western blotting analysis of remaining NeuN protein levels after CRISPR-mediated disruption. In agreement with the results obtained from the cell lines (Fig. 1), sgNeuN#2 and sgNeuN#3 depleted endogenous NeuN protein by ~82–93% in primary hippocampal neuron culture, regardless of the scaffolds used (Fig. 2b). Consistently, sgNeuN#2 and sgNeuN#3 reduced NeuN expression by ~87–90% in primary cortical neuron culture (Fig. 2c), with sgNeuN#2 being slightly more potent overall (Fig. 2). We observed ~37% reduction of NeuN proteins by treatment with sgNeuN#1-BH, whereas only ~15% reduction of NeuN was observed upon treatment with sgNeuN#1-FZ, suggesting the BH scaffold can elicit a greater potency than the FZ scaffold for a less effective spacer (Fig. 2b). Although the difference is modest, the BH scaffold was utilized in the subsequent in vivo studies.

**Fig. 3:**
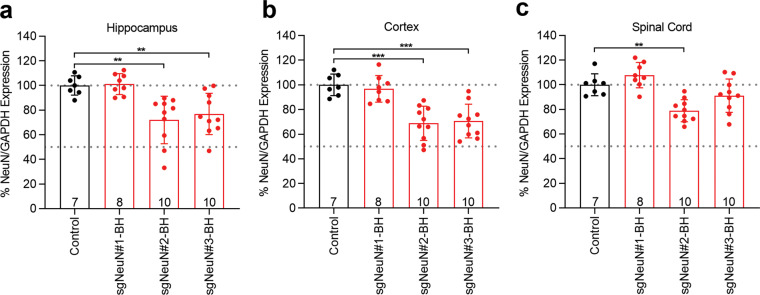
NeuN gene disruption led to sub-optimal NeuN reduction in the CNS of mice. AAV9-sgNeuN#2-BH and AAV9-sgNeuN#3-BH significantly reduced NeuN expression in the **a** hippocampus, **b** cortex, and **c** spinal cord. AAV9-sgNeuN#1–3-BH (4.3 × 10^10^ GC) were injected into P0 pups via ICV. CNS tissues were harvested at 6 weeks of age for protein quantification. Data represent means ± SD of *n* samples noted above the *x*-axis and significance was tested with one-way ANNOVA followed by Dunnett’s multiple comparison test; **P < 0.01, ***P < 0.001. Refer to Fig. S6–S8 for representative gel images.

Finally, we examined the relative potency of spacer sequences #1–#3 paired with the BH scaffold in disrupting endogenous NeuN expression in mouse CNS. ICV injections were used to deliver AAV9-sgRNAs directly into the CSF of neonatal mice at postnatal day 0 (P0) to enable widespread transduction in the CNS [36, 37]. Among the three spacer sequences, only sgNeuN#2-BH reliably led to significant reduction of NeuN proteins by ~22–32%, following ICV injection of AAV9 (4.3 × 10^10^ GC), in all three CNS regions examined: hippocampus, cortex and spinal cord (one-way ANNOVA followed by Dunnett’s multiple comparison test; **P < 0.01, ***P < 0.001) (Fig. 3a–c). In contrast, sgNeuN#1 failed to result in any measurable NeuN reduction, and sgNeuN#3-BH resulted in slight but not statistically significant reduction of NeuN in the spinal cord (Fig. 3c). Taken together, these results support the validity of using the in vitro triage described above to predict relative in vivo CRISPR activity of the sgRNA designs.

### Neonatal ICV delivery of CRISPR-sgRNA in vivo by AAV-PHP.B achieves the prominent reduction of NeuN levels in mouse CNS

**Fig. 4:**
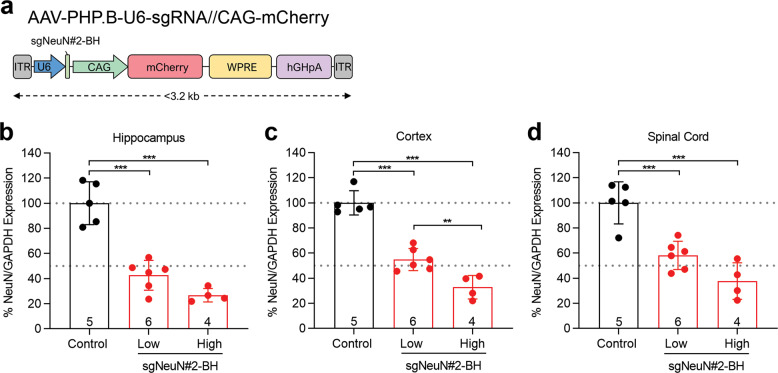
Injection of AAV-PHP.B-sgNeuN#2-BH significantly decreased NeuN protein level in the brain. **a** Vector map of the AAV-PHP.B transgene. Injection of the high dose (2 × 10^11^ GC) further decreased NeuN protein levels relative to the low dose (1 × 10^11^ GC) injections in the **b** hippocampus, **c** cortex, and **d** spinal cord. AAV-PHP.B-sgNeuN#2-BH was bilaterally injected into P0 pups via ICV. CNS tissues were harvested at 6 weeks of age for protein quantification. All data points represent means ± SD of *n* samples noted above the *x*-axis and significance was tested with one-way ANNOVA followed by Tukey’s multiple comparison test; **P < 0.01, ***P < 0.001. Refer to Fig. S9–S11 for representative gel images.

Next, we sought to further enhance the degree of the NeuN knockout in vivo by adjusting the parameters used to deliver AAV in the CNS, such as the dosage and the capsid (Torregrosa et al. 2021, in review [38]). AAV-PHP.B capsid was employed, since it has been reported to result in at least ×40 greater transduction than AAV9 in neurons and astrocytes across different CNS regions in C57BL/6J mice, despite much reduced CNS transduction in other mouse strains and non-human primates [19, 39]. Neonatal Cas9 mice in C57BL/6J background were ICV injected with AAV-PHP.B containing sgNeuN#2-BH at two dose levels. Injection of AAV-PHP.B-sgNeuN#2-BH at 1 × 10^11^ GC achieved ~39–58% of NeuN protein reduction (Fig. 4a–d). When the dosage was increased to 2 × 10^11^ GC, greater NeuN protein reduction was achieved in the CNS, ranging from ~63–74% (Fig. 4b–d). Maximal NeuN reduction was observed in the hippocampus at ~74% (Fig. 4b). In general, neonatal ICV injection of greater number of viral particles for up to 3.2 × 10^11^ tested improved NeuN reduction and was well tolerated in mice without causing tissue injury or cell death (data not shown). Thus, the use of AAV-PHP.B at higher doses can dramatically enhance CRISPR-mediated NeuN gene disruption and subsequent protein reduction in bulk CNS tissues.

### Immunohistochemical analysis confirms robust NeuN gene disruption in multiple CNS subregions

Next, to confirm high neuronal transduction in the CNS by AAV-PHP.B at a higher dose level, we used immunohistochemical (IHC) staining to directly visualize and quantify expression of the reporter gene mCherry. Neonatal Cas9 mice were ICV injected with AAV-PHP.B encoding for mCherry at 2 × 10^11^ GC and were then sacrificed at 6 weeks after injection for IHC staining and analysis. Neuronal transduction was estimated by quantifying the percentage of mCherry-expressing neurons stained positive for NeuN (NeuN^+^) among all the NeuN^+^ neurons in the images analyzed (Table S3). Overall, high rate of neuronal transduction was observed in the hippocampus, cortex, dorsal and ventral spinal cord, ranging from 57 to 97% (Fig. S12).

**Fig. 5:**
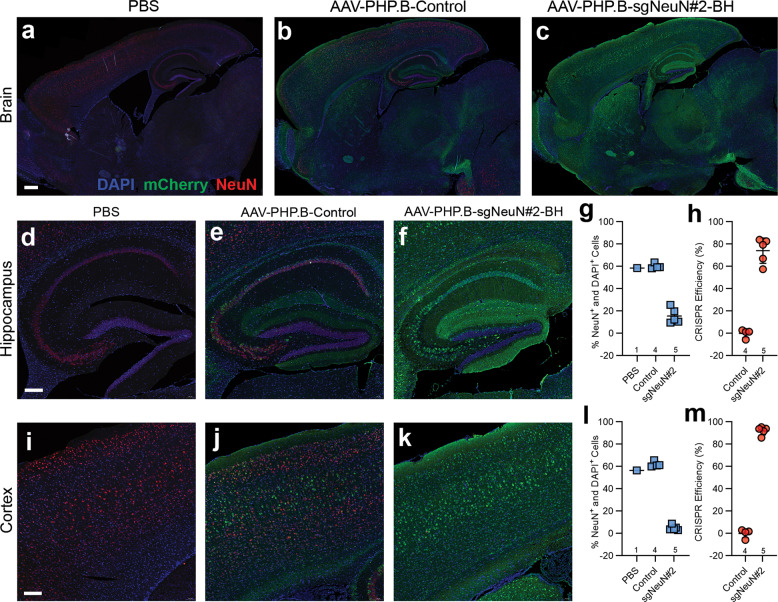
Immunohistochemical analysis confirms disruption of NeuN in the brain. **a**–**c** General NeuN signal reduction is detected in the hippocampus and the cortex. **d**–**f** AAV-PHP.B-sgNeuN#2-BH led to a decrease in NeuN in the CA1 (Cornu Ammonis), CA2 and CA3 regions of the hippocampus. **g**, **h** Quantification analysis showing robust CRISPR knockdown efficiency in hippocampal neurons. **i**–**k** Sharp reduction of NeuN expression was detected in the superficial and deep regions of the cortex. **l**, **m** AAV-PHP.B-sgNeuN#2-BH achieves a high CRISPR efficiency in the cortex. Brain tissues were collected and fixed for double-immuno-staining with anti-NeuN (red) and anti-mCherry (green) antibodies. Data represent means ± SD of *n* samples noted above the x-axis. Scale bars: (**a**–**c**) = 500 μm, (**d**–**f**) = 200 μm, (**i**–**k**) = 100 μm. (color figure online).

**Fig. 6:**
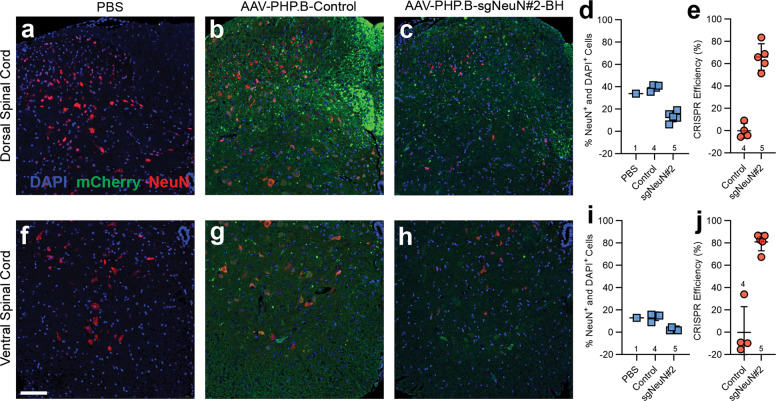
Immunohistochemical staining reveals robust NeuN gene disruption in the spinal cord. **a**–**c** A notable decrease in the NeuN signal is detected in the dorsal neurons of the spinal cord. **d**, **e** Neuronal presence and CRISPR efficiency in the dorsal region of the spinal cord. **f**–**h** Cell distribution of NeuN positive cells post AAV-PHP.B-sgNeuN#2-BH treatment in the ventral region of the spinal cord. **i**, **j** Neuronal presence and CRISPR efficiency in the ventral region of the spinal cord. The spinal cord was fixed, embedded, and sectioned for double immune-staining for NeuN (red) and mCherry (green). Data represent means ± SD of *n* samples noted above the *x*-axis. Scale bars: (**a**–**c**, **f**, **g**) = 100 μm. (color figure online).

In addition, we assessed the efficiency of CRISPR-mediated neuronal gene disruption at the cellular level by comparing the number of neurons stained positive for NeuN proteins in mice injected with AAV-PHP.B-sgNeuN#2-BH versus mice injected with AAV-PHP.B-Control. DAPI staining (DAPI^+^) was used to label the nuclei of all the cells in the images analyzed. Percentage of NeuN^+^ neurons among all the cells (DAPI^+^) were analyzed (Table S3) and indicated that ~60% of NeuN^+^ neuronal populations were present in the hippocampus and the cortex (Fig. 5g, l), while ~34% and ~13% of NeuN^+^ neuronal populations were present in the dorsal and the ventral spinal cord, respectively (Fig. 6d, i). Following viral delivery of sgNeuN#2-BH, percent NeuN^+^ neuronal populations notably diminished in all four CNS regions analyzed, suggesting prominent disruption of NeuN expression by CRISPR (Fig. 5g, l; Fig. 6d, i). CRISPR efficiency was calculated by the percent decrease of NeuN^+^ neuronal population from the control mice to sgNeuN#2-BH treated mice (Table S4). As a result, AAV-PHP.B-sgNeuN#2 achieved >90% CRISPR efficiency in the cortex (Fig. 5i–m) and led to a CRISPR efficiency of 74.1 ± 11.4% in the hippocampus (Fig. 5d–h), 66.1 ± 11.8% in the dorsal spinal cord (Fig. 6e) and 81.1 ± 7.9% in the ventral spinal cord (Fig. 6j). Qualitative examination of other brain regions revealed differential reduction of NeuN by CRISPR, corresponding to varied neuronal transduction. In the cerebellum, AAV-PHP.B transduced Purkinje cells displaying prominent mCherry signals in the cell body layer and in the dendrites extending into the molecular layer (Fig. S13a–c). However, cerebellar granule cells were poorly transduced, and a strong NeuN signal was observed in the densely packed granular layer of the cerebellar cortex, due to the lack of NeuN gene disruption by sgNeuN#2-BH (Fig. S13b, c). In the pons, NeuN signal clearly diminished by CRISPR NeuN knockout, despite weaker mCherry signals (Fig. S13d–f). Although the baseline NeuN signal was generally lower in the striatum relative to other CNS regions (Fig. S13g–i), we observed an unambiguous decrease in NeuN signal in mice treated with AAV-PHP.B-sgNeuN#2-BH. Overall, these results demonstrated that apart from the cerebellar granule cells, the CRISPR-sgNeuN#2-BH delivered by AAV-PHP.B achieves broad NeuN gene editing in the CNS.

### AAV-PHP.B-sgNeuN#2-BH leads to biallelic indels in the cortex

**Fig. 7:**
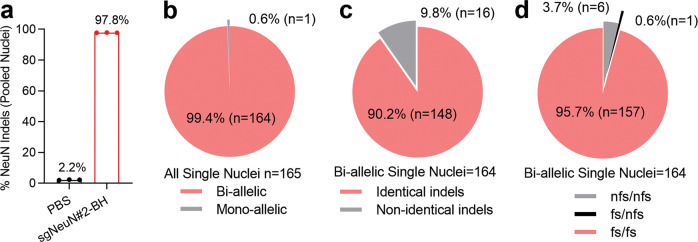
CRISPR-Cas knockout of the NeuN gene achieves biallelic frameshift indels in the cortex. **a** The NeuN indel rate in all pooled nuclei dissociated from transduced cells. **b** Almost all examined single nuclei showed biallelic NeuN indels. **c** The type of biallelic indels detected, the vast majority are identical indels indicating homogenous sgNeuN#2-BH activity. **d** The breakdown of the type of indels regardless if identical or not, frameshift mutations in both alleles are the most prominent type of NeuN gene disruption. Nuclei were dissociated, FACs sorted and NGS sequenced for indel analysis. A total of *n* = 3 mice were used for sequencing. fs frameshift, nfs non-frameshift.

-BH. Neonatal Cas9 mice were injected with AAV-PHP.B-sgNeuN#2-BH vector containing eGFP along with the KASH domain which tethers the eGFP proteins at the nuclear membrane (Table S1). Cortices were collected 5 weeks post injection for nuclei dissociation, FACS sorting and NGS sequencing at the NeuN locus. In pooled nuclei, ~97.8% of the NeuN amplicons contained indels, including insertions and deletions, suggesting high NeuN gene editing efficiency (Fig. 7a). Of the 165 single cortical nuclei analyzed, 164 nuclei contained indels in both NeuN alleles, representing ~99.4% biallelic gene editing rate (Fig. 7b). Furthermore, ~90.2% of single nuclei containing biallelic editing exhibited identical indels in both alleles indicating a rather homogeneous indel profile (Fig. 7c). Of the 164 biallelic nuclei, 157 nuclei contained indels in both alleles that led to frameshift mutations (shift of 3n + 1 or 3n + 2), indicating complete disruption of NeuN expression in ~95.7% of all biallelic nuclei (Fig. 7d). In conclusion, this data suggested that CRISPR-mediated gene editing of NeuN in vivo can lead to an outcome of complete gene knockout at the single cell level in the CNS.

## Discussion

Systematic comparison of sgNeuN spacer sequences and sgRNA scaffolds in multiple in vitro cell models identified sgNeuN#2-BH as the most efficient sgRNA, which also led to maximal NeuN reduction in vivo (Figs. 1, 2, and 3). The reduction of NeuN by the sgRNA in vivo was initially limited due to modest neuronal transduction by AAV9. However, the use of a more efficient capsid, AAV-PHP.B, along with neonatal ICV administration at a higher dose level markedly decreased NeuN protein levels by ~63–74% in the bulk CNS tissues (Figs. 3 and 4). Immunohistochemistry analysis in the brain and the spinal cord of the ICV injected mice also showed that very few neurons stained positive for NeuN proteins across multiple CNS subregions, demonstrating the ability of the CRISPR approach to achieve remarkably broad neuronal gene knockout in vivo. (Figs. 5 and 6). Further profiling of the indels revealed over ~99.4% rate of biallelic editing of the NeuN gene, suggesting highly efficient neuronal gene knockout at single cell level (Fig. 7). Interestingly, we observed rather homogenous genomic edits introduced by sgNeuN#2-BH, indicating the behavior of an sgRNA in vivo is not totally stochastic. It is worth noting that CRISPR-Cas9-mediated reduction of NeuN was strikingly effective at ≥90% in primary neuron cultures, a cell model commonly used to study the molecular basis of neuronal signaling and synaptic plasticity. This result is in agreement with previous studies examining CRISPR knockout of two proteins in primary cortical and hippocampal neurons [40, 41].

Enhancing the CRISPR activity and achieving higher knockout efficiency required the selection of an optimal spacer and sgRNA scaffold combination. Among the three spacer sequences, sgNeuN#2 was shown to consistently lead to maximal NeuN protein reduction in multiple in vitro systems as well as in mice, indicating that in vitro systems can closely predict the in vivo performance of an sgRNA. Several avenues have been pursued to improve the sgRNA scaffold, such as lengthening the sgRNA hairpin structure by 5 bp [42]. Chen et al. optimized the most commonly used sgRNA scaffold, the FZ scaffold, by further extending the hairpin Cas9 binding structure and by removing the termination sequence, A-U flip, in the crRNA direct repeats sequence [14]. Elimination of the stop sequence is predicted to increase sgRNA transcription and extension of the hairpin structure by five nucleotides increases Cas9 binding to the sgRNA [14, 42]. These changes to the sgRNA scaffold, referred to the BH in this study, enhanced activity and increased dCas9-EGFP imaging efficiency [14]. Indeed, the results from our cell-based assays revealed that the BH scaffold exerted greater CRISPR activity than the FZ scaffold in mediating gene disruption. The BH scaffold’s superiority is most notable when it is combined with a less effective spacer sequence—sgNeuN#1. When combined with the more effective spacers #2 and #3, no significant improvement was observed by the BH scaffold. This indicated that the overall effectiveness of Cas9-mediated gene disruption may hinge more on the spacer sequence and less on the sgRNA scaffold sequence.

The level of NeuN protein remaining in the CNS tissues analyzed via western blotting was relatively consistent with immunohistochemistry in mice. Western blotting is more accurate than IHC in quantifying protein levels. Immunohistochemistry is capable of semi-quantitatively measuring gene knockout at the cellular level [43]. Dittadi et al. has previously compared these methods and reported that western blot and IHC results are in agreement for cells which express high levels of the target proteins [44]. In this study, western blot results showed that Cas9-mediated NeuN reduction was more prominent in the cortex and the hippocampus than in the spinal cord. Consistently, IHC results also suggested that CRISPR efficiency is the highest in the cortex and the hippocampus among several CNS regions examined. In addition, other regions of the brain, such as the cerebellum, showed differential NeuN and mCherry immunostaining. The Purkinje cell body layer and the dendrites in the molecular layer of the cerebellum were transduced. However, in the granule cell layer, mCherry’s signal was low due to poor transduction and strong NeuN signal remained. Western blotting analysis also confirmed lack of NeuN disruption in the cerebellum (data not shown). Our observations are in accordance with many published studies utilizing IV or ICV to administer AAV9 and AAV-PHP.B in neonate and adult mice [18, 19, 45–48]. These studies reported varying level of purkinje cell transduction and very minimal transduction of the granule cells in the cerebellum. Interestingly, transduction of the cerebellar granule cells is shown successful when AAV is delivered via intraparenchymal or intracisternal magna (ICM) injections [45, 49, 50]. Additional studies will be needed to determine if effective gene knockout can be achieved in the cerebellum when sgRNA is delivered by AAV following intraparenchymal or ICM injections.

In our study, Cas9-mediated NeuN knockout achieved 99.4% biallelic indels of which 95.7% led to frameshift mutations in both alleles in the cortex. This is higher than the NeuN indel rate reported by Platt et al. where NeuN indels represented 84% biallelic mutations [10]. These improvements could possibly be accounted by the improved spacer as well as the scaffold of the sgRNA [12, 14]. It is worth noting that in Platt et al., NeuN knockout was only achieved locally at the site of injection, whereas we achieved broad NeuN knockout in the CNS with P0 ICV. However, additional experiments will need to be conducted to examine the potential off-target effects by the sgRNA used in this study. Off-target effects by CRISPR continues to be an important concern in the field and a number of studies have developed methodologies to examined it [51–53]. In addition, the efficiency of CRISPR in Cas9 knock-in mice is superior to a system with ectopic expression of Cas9 [54], as evident by the much reduced indel rate observed when *Staphylococcus aureus* Cas9 is packaged along with the sgRNA in an AAV vector [54]. Dual vector delivery strategy employing two AAVs for the Cas9 and the sgRNA also led to lower indel rate, as reported by Swiech et al. (67.5%) in comparison with ~99% indel rate observed in our study [41]. Also, the efficiency of the dual vector strategy is limited, ornithine transcarboxylase deficient mice treated two AAVs resulted in reversion of the ornithine transcarboxylase mutation in 10% of hepatocytes [55]. The expression of Cas9 in the knock-in mice is broad and sustained whereas the expression of Cas9 in the dual AAV strategy is restricted to the transduced cells and could decline overtime upon silencing of the promoter in the vector genome [55]. In conclusion, our CRISPR-Cas method (1) allows for the rapid generation of broad gene knockout in murine CNS, (2) is more efficient than the dual AAV strategies for Cas9 delivery, (3) enables gene disruption at postnatal stage and thereby avoids potential lethality associated with knockout of a gene essential for embryonic development, and (4) is relatively simpler and faster than the traditional gene knockout methods using embryonic stem cells. We believe this study provided a new and easy way for researchers to generate murine knockout models which should facilitate neurobiology reserach and the discovery of mechanisms underlying CNS diseases.

## Supplementary information

Supplementary Material
