# Supplementary material for: Highly efficient neuronal gene knockout in vivo by CRISPR-Cas9 via neonatal intracerebroventricular injection of AAV in mice
Source: Gene Ther. 2021 Feb 8;28(10-11):646–58. doi: 10.1038/s41434-021-00224-2 (PMC8599009; doi:10.1038/s41434-021-00224-2)
Supplement: Supplementary file 1 — Supplementary Material [file 41434_2021_224_MOESM1_ESM.docx]

***Supplementary Material***

Efficient neuronal gene knockout *in vivo* by CRISPR-Cas9 via neonatal intracerebroventricular injection of AAV in mice

Sam Hana^1^, Michael Peterson^1^, Olivia McKissick^1^, Helen McLaughlin^1^, Eric Marshall^1^, Attila J. Fabian^1^, Kathryn Koszka^1^, Galina Marsh^1^, Michael Craft^1^, Shanqin Xu^1^, Alexander Sorets^1^, Tess Torregrosa^1^, Chao Sun^1^, Chris E. Henderson^1^, Shih-Ching Lo^1^

^1^Biogen Inc., Cambridge, Massachusetts, United States of America.

Keywords: CRISPR, neonatal ICV, knockout, AAV, NeuN, Indel

*Correspondence should be addressed to:

Shih-Ching Lo, 225 Binney Street, Cambridge, MA 02142 USA, Email: joyce.lo@biogen.com

**
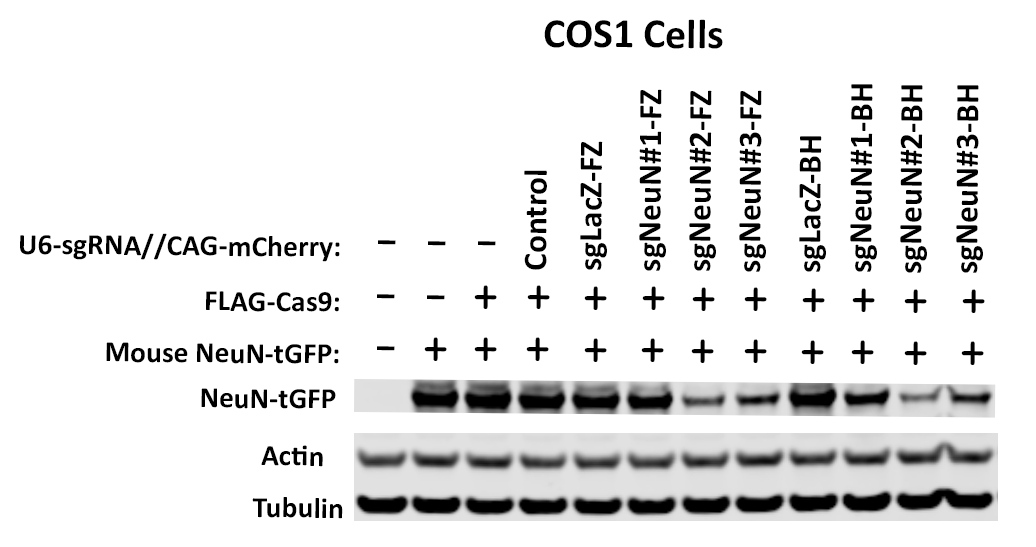
**

**Fig S1. Western blot showing the effect of different spacer sequences, #1, #2 or #3, and sgRNA backbones, FZ or BH, on CRISPR-mediated reduction of exogenous NeuN protein levels in COS1 cells. For controls, either sgRNA was omitted (Control) or an sgRNA targeting the LacZ gene was used.**

**
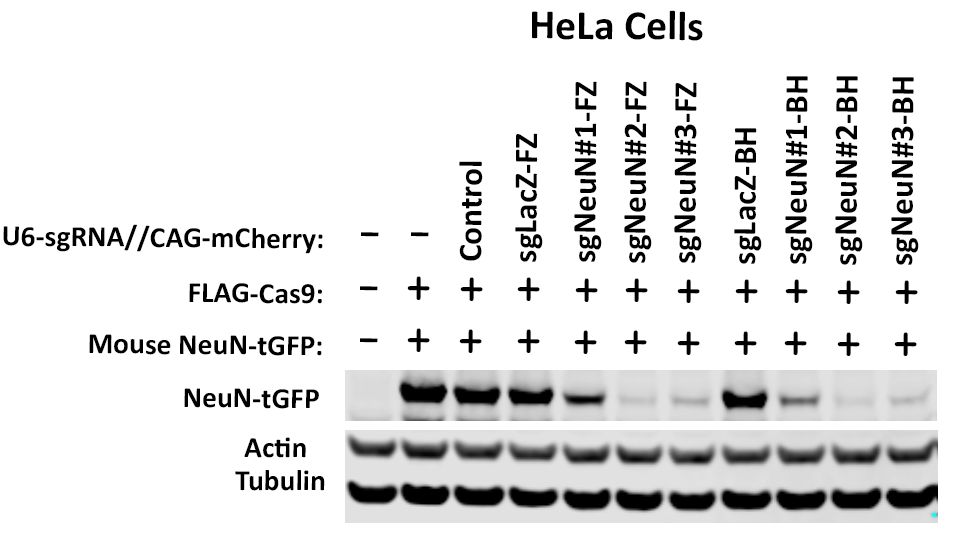
**

**Fig S2. Western blot showing the effect of different spacer sequences, #1, #2 or #3, and sgRNA backbones, FZ or BH, on CRISPR-mediated reduction of exogenous NeuN protein levels in HeLa cells stably expressing spCas9.** **For controls, either sgRNA was omitted (Control) or an sgRNA targeting the LacZ gene was used.**


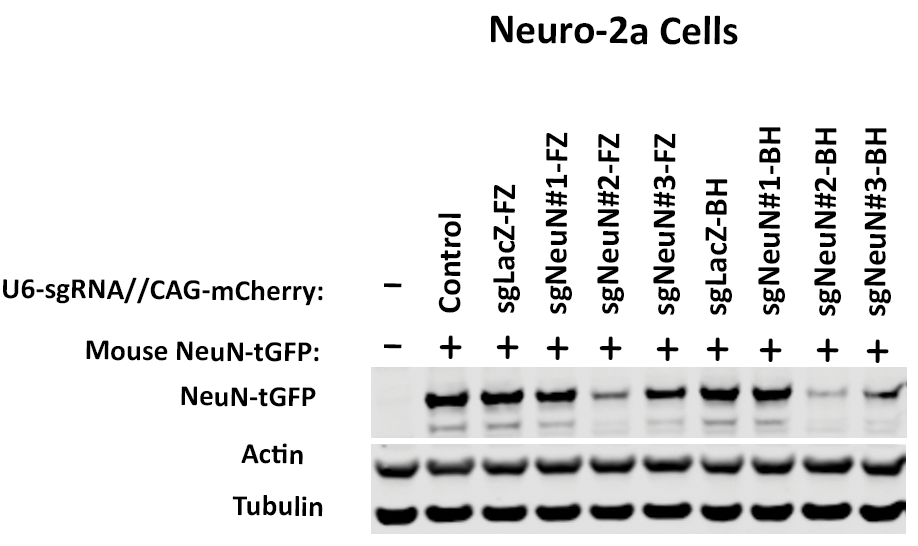


**Fig S3. Western blot showing the effect of different spacer sequences, #1, #2 or #3, and sgRNA backbones, FZ or BH, on CRISPR-mediated reduction of exogenous NeuN protein levels in Neuro-2a cells induced to expressed spCas9 by doxycycline.** **For controls, either sgRNA was omitted (Control) or an sgRNA targeting the LacZ gene was used.**


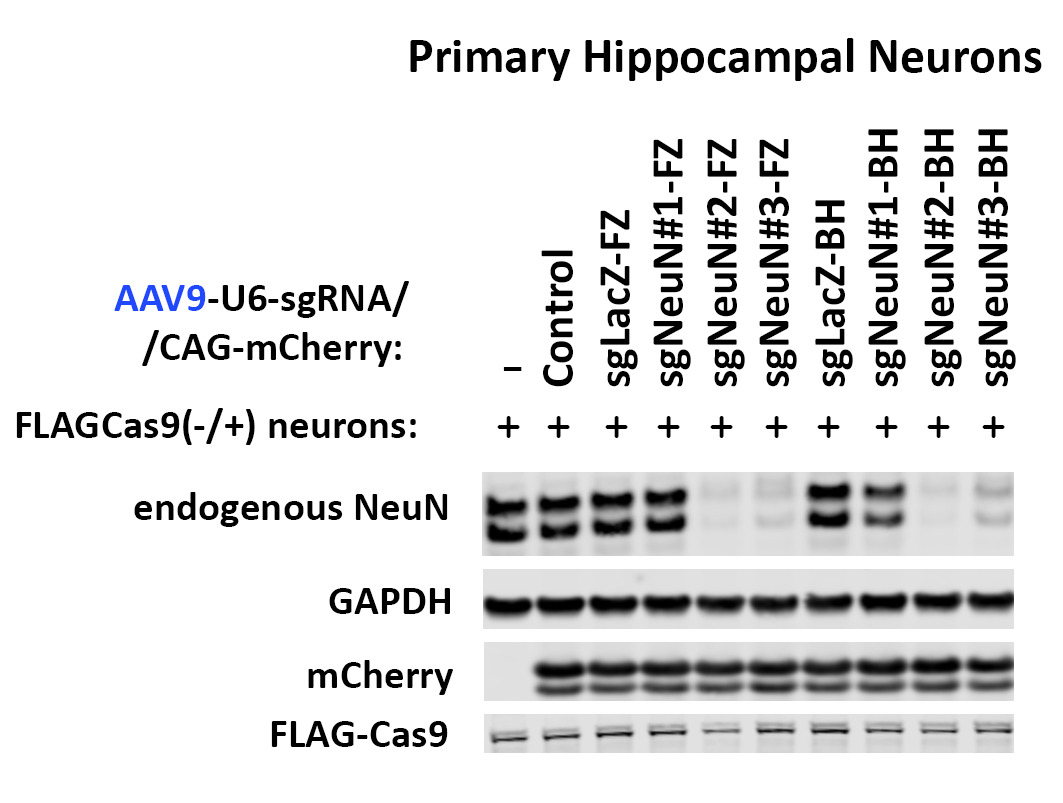


**Fig S4. Western blot showing the effect of different spacer sequences, #1, #2 or #3, and sgRNA backbones, FZ or BH, on AAV9-CRISPR-mediated reduction of endogenous NeuN protein levels in primary mouse hippocampal neuron culture prepared from Cas9^-/+^ mouse embryos. For controls, either sgRNA was omitted (Control) or an sgRNA targeting the LacZ gene was used.**


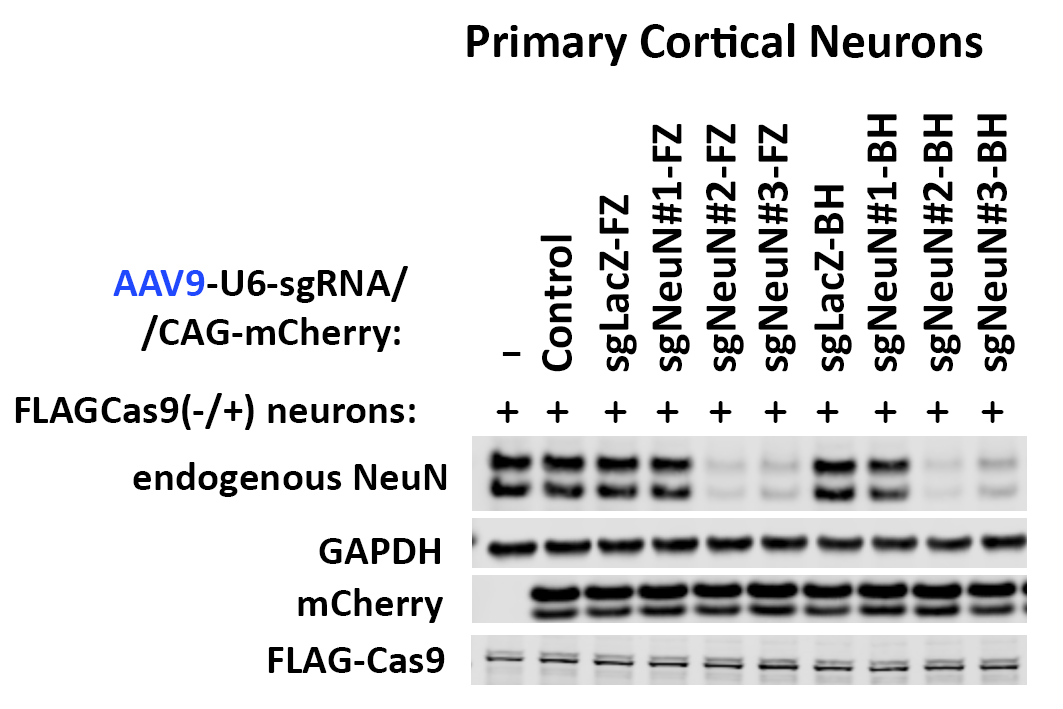


**Fig S5. Western blot showing the effect of different spacer sequences, #1, #2 or #3, and sgRNA backbones, FZ or BH, on AAV9-CRISPR-mediated reduction of endogenous NeuN protein levels in primary mouse cortical neuron culture prepared from Cas9^-/+^ mouse embryos. For controls, either sgRNA was omitted (Control) or an sgRNA targeting the LacZ gene was used.**


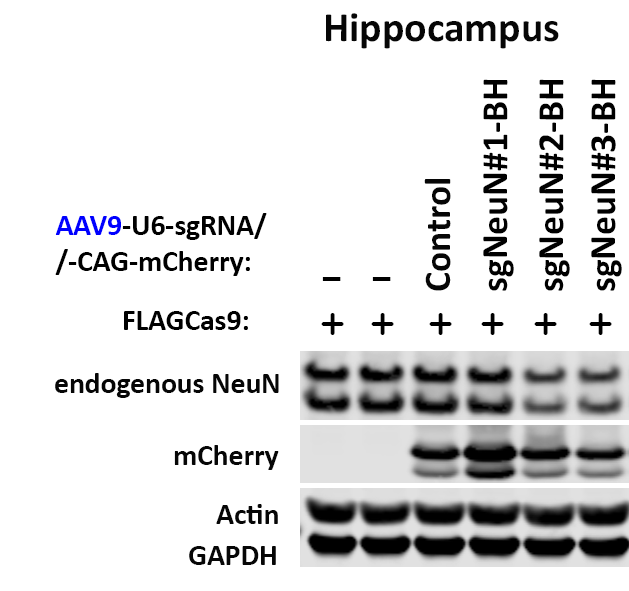


**Fig S6. Sample western blot showing the effect of different spacer sequences, #1, #2 or #3, on AAV9-CRISPR-mediated reduction of endogenous NeuN protein levels in the hippocampus of 6 weeks of age mice. The Control treatment lacks a coding sgRNA.**


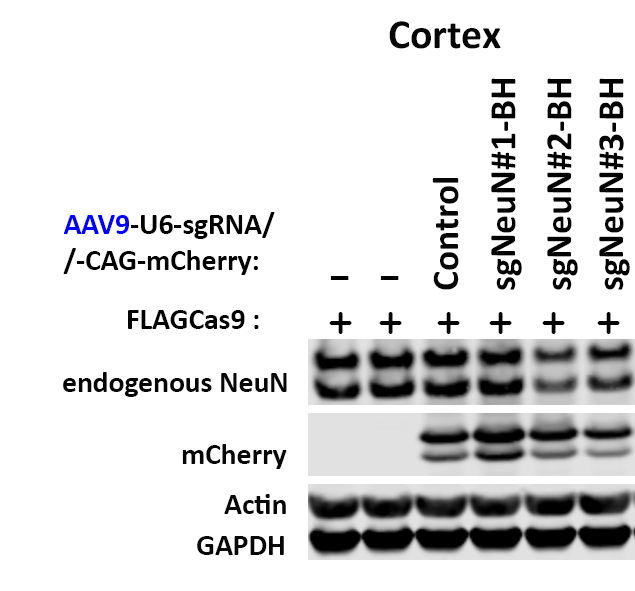


**Fig S7. Sample western blot showing the effect of different spacer sequences, #1, #2 or #3, on AAV9-CRISPR-mediated reduction of endogenous NeuN protein levels in the cortex of 6 weeks of age mice. The Control treatment lacks a coding sgRNA.**


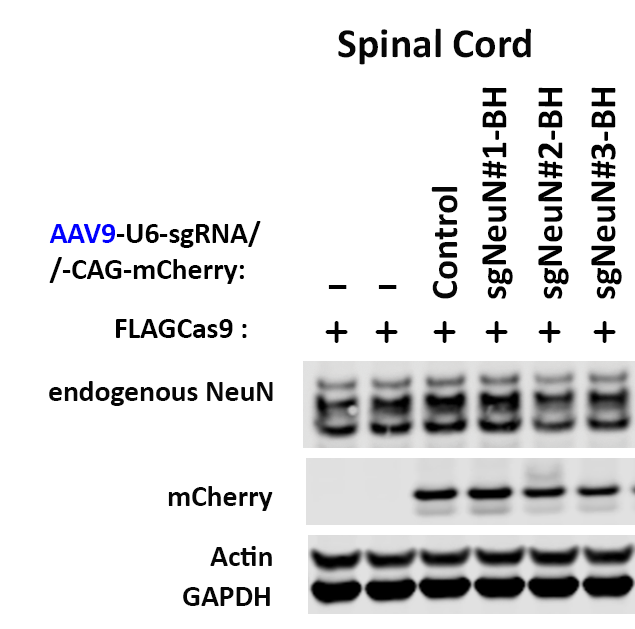


**Fig S8. Sample western blot showing the effect of different spacer sequences, #1, #2 or #3, on AAV9-CRISPR-mediated reduction of endogenous NeuN protein levels in the spinal cord of 6 weeks of age mice. The Control treatment lacks a coding sgRNA.**


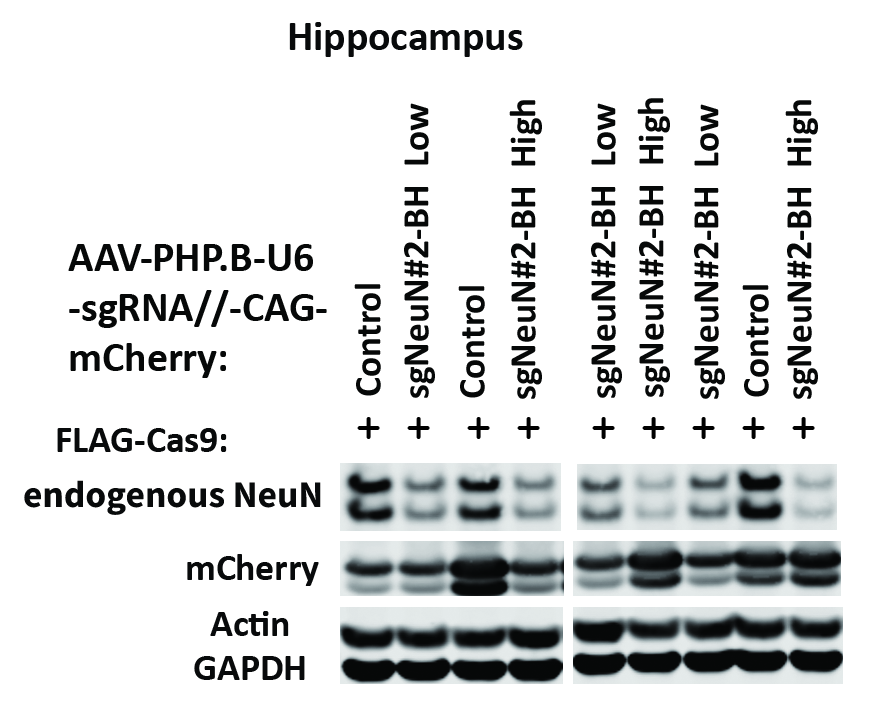


**Fig S9. Sample western blot showing the effect of different dose levels of AAV-PHP.B on CRISPR-mediated reduction of endogenous NeuN protein levels in the hippocampus of 6 weeks of age mice. The Control treatment lacks a coding sgRNA.**


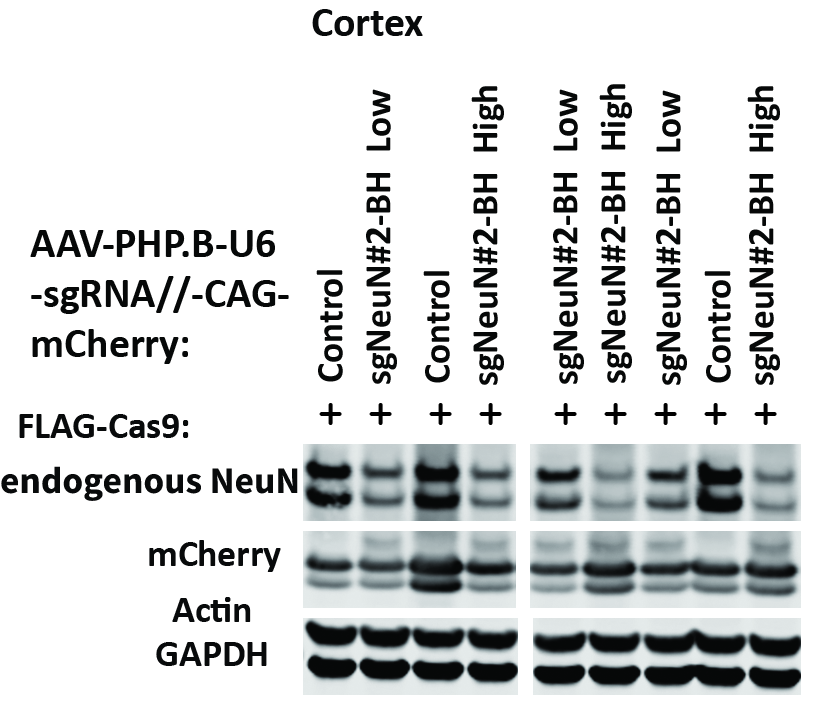


**Fig S10. Sample western blot showing the effect of different dose levels of AAV-PHP.B on CRISPR-mediated reduction of endogenous NeuN protein levels in the hippocampus of 6 weeks of age mice. The Control treatment lacks a coding sgRNA.**


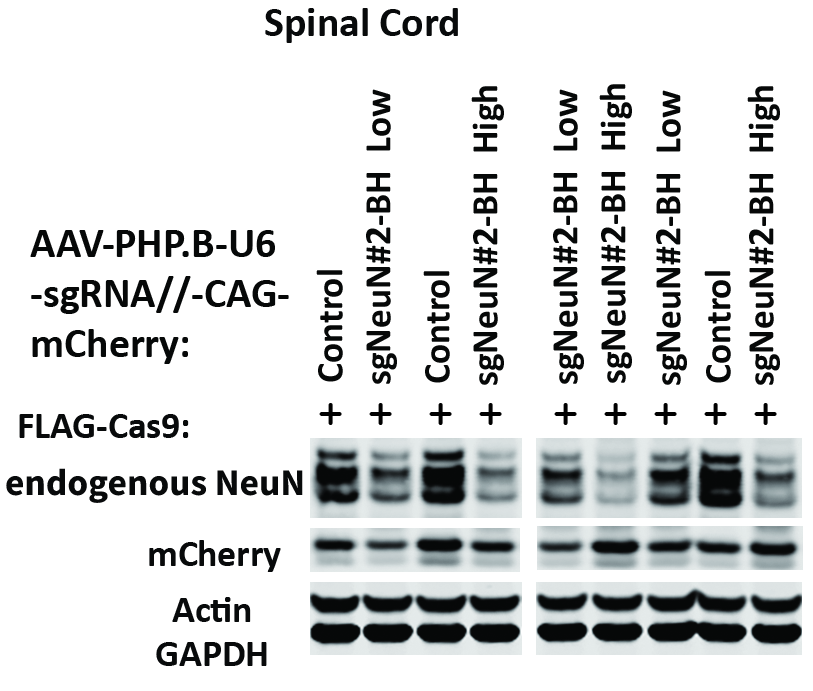


**Fig S11. Sample western blot showing the effect of different dose levels of AAV-PHP.B on CRISPR-mediated reduction of endogenous NeuN protein levels in the spinal cord of 6 weeks of age mice. The Control treatment lacks a coding sgRNA.**


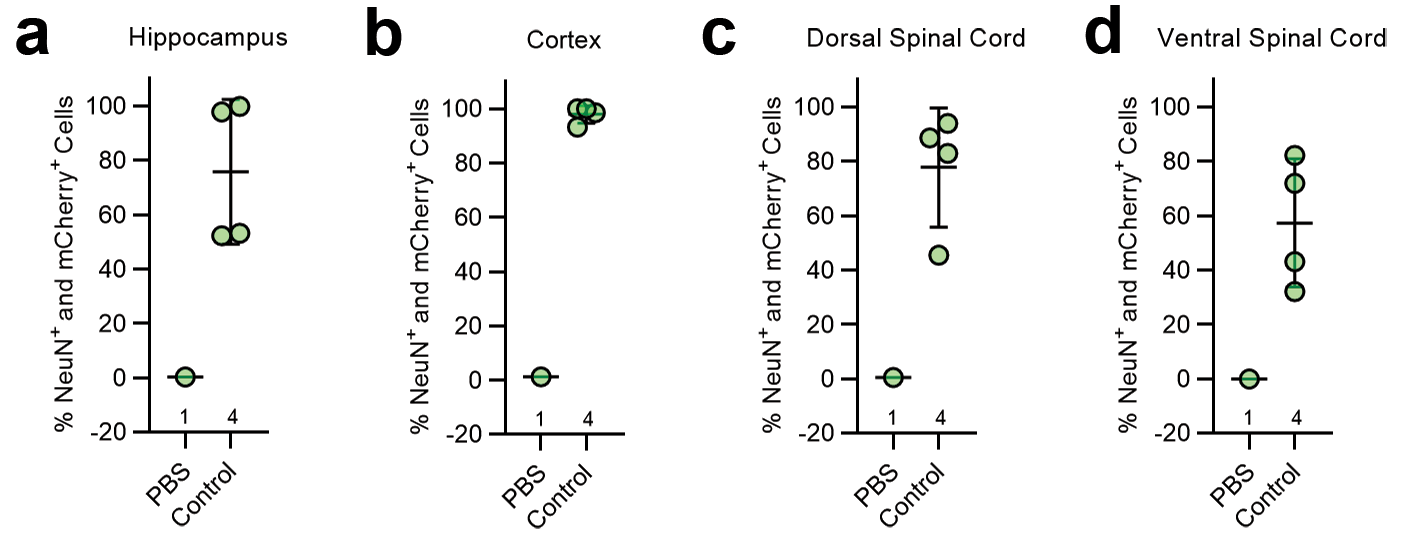


**Fig S12. AAV-PHP.B mediates good neuronal transduction efficiency in the brain and the spinal cord following neonatal ICV injection.** The percent NeuN positive and mCherry positive cells in the **(a)** hippocampus, **(b)** cortex, **(c)** dorsal spinal cord, **(d)** ventral spinal cord. Data points represent means ± SD of *n* samples noted above the x-axis.


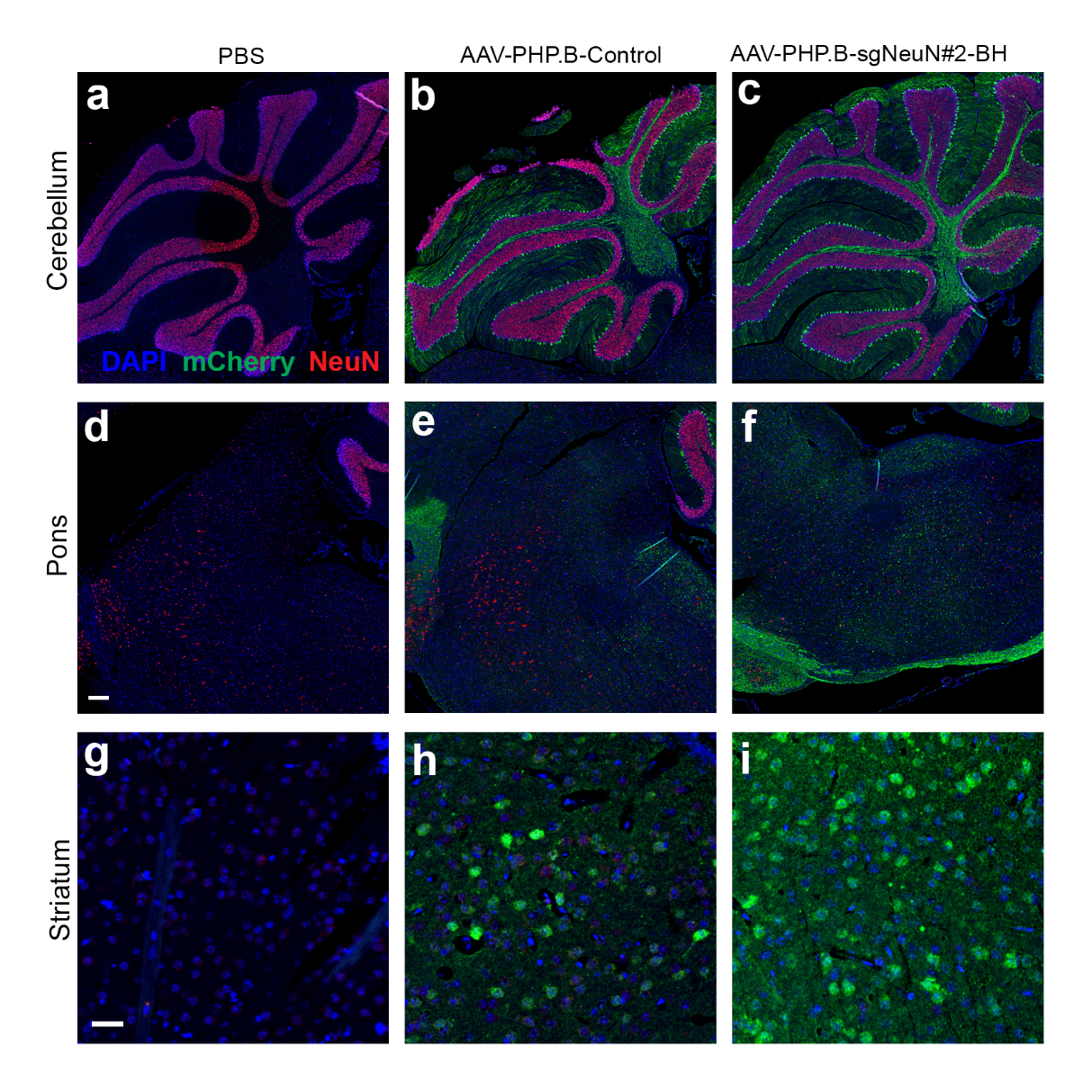


**Fig S13. Immunohistochemical analysis of other brain regions shows variable CRISPR-Cas9 knockout of NeuN**. **(a-c)** Lack of transduction and NeuN knockout in the granular cells of the cerebellum. **(d-f)** Fair reduction of NeuN in the pons. **(g-i)** The striatum images are at a higher magnification to note the low NeuN signal. Brain tissues were dissected and fixed for double-immuno-staining with anti-NeuN (red) and anti-mCherry (green) antibodies. Scale bars: (a-f) = 300 μm**,** (g-i) = 40 μm

**Table S1. A list of all plasmids, AAV constructs and primers used in the study.**

| **#** | **Reagent** | **Type** | **Use** |
| --- | --- | --- | --- |
| **1** | pFB-U6-sgNeuN#1-FZ//CAG-mCherry-WPRE-hGHpA | Plasmid | In vitro validation (Fig. 1) |
| **2** | pFB-U6-sgNeuN#2-FZ//CAG-mCherry-WPRE-hGHpA | Plasmid | In vitro validation (Fig. 1) |
| **3** | pFB-U6-sgNeuN#3-FZ//CAG-mCherry-WPRE-hGHpA | Plasmid | In vitro validation (Fig. 1) |
| **4** | pFB-U6-sgLacZ-FZ//CAG-mCherry-WPRE-hGHpA | Plasmid | In vitro validation (Fig. 1) |
| **5** | pFB-U6-CAG-mCherry-WPRE-hGHpA (termed Control) | Plasmid | In vitro validation (Fig. 1) |
| **6** | pFB-U6-sgNeuN#1-BH//CAG-mCherry-WPRE-hGHpA | Plasmid | In vitro validation (Fig. 1) |
| **7** | pFB-U6-sgNeuN#2-BH//CAG-mCherry-WPRE-hGHpA | Plasmid | In vitro validation (Fig. 1) |
| **8** | pFB-U6-sgNeuN#3-BH//CAG-mCherry-WPRE-hGHpA | Plasmid | In vitro validation (Fig. 1) |
| **9** | pFB-U6-sgLacZ-BH//CAG-mCherry-WPRE-hGHpA | Plasmid | In vitro validation (Fig. 1) |
| **10** | pVAX1-FLAG-[NLS]spCas9[NLS] | Plasmid | In vitro validation (Fig. 1) |
| **11** | pCMV6-Mm.NeuN(Rbfox3)-turboGFP-hGHpA | Plasmid | In vitro validation (Fig. 1) |
| **12** | AAV9-U6-sgNeuN#1-FZ//CAG-mCherry-WPRE-hGHpA | Viral Vector | In vitro and in vivo validation (Fig. 2) |
| **13** | AAV9-U6-sgNeuN#2-FZ//CAG-mCherry-WPRE-hGHpA | Viral Vector | In vitro and in vivo validation (Fig. 2) |
| **14** | AAV9-U6-sgNeuN#3-FZ//CAG-mCherry-WPRE-hGHpA | Viral Vector | In vitro and in vivo validation (Fig. 2) |
| **15** | AAV9-U6-sgLacZ-FZ//CAG-mCherry-WPRE-hGHpA | Viral Vector | In vitro and in vivo validation (Fig. 2) |
| **16** | AAV9-U6-CAG-mCherry-WPRE-hGHpA (termed Control) | Viral Vector | In vitro and in vivo validation (Fig. 2) |
| **17** | AAV9-U6-sgNeuN#1-BH//CAG-mCherry-WPRE-hGHpA | Viral Vector | In vitro and in vivo validation (Fig. 2, 3) |
| **18** | AAV9-U6-sgNeuN#2-BH//CAG-mCherry-WPRE-hGHpA | Viral Vector | In vitro and in vivo validation (Fig. 2, 3, 4) |
| **19** | AAV9-U6-sgNeuN#3-BH//CAG-mCherry-WPRE-hGHpA | Viral Vector | In vitro and in vivo validation (Fig. 2, 3) |
| **20** | AAV9-U6-sgLacZ-BH//CAG-mCherry-WPRE-hGHpA | Viral Vector | In vitro and in vivo validation (Fig. 2, 3) |
| **21** | AAV-PHP.B-U6-Control//CAG-mCherry-WPRE-hGHpA | Viral Vector | In vivo characterization by immunoblotting and IHC (Fig. 5, 6) |
| **22** | AAV-PHP.B-U6-sgNeuN#2-BH//CAG-mCherry-WPRE-hGHpA | Viral Vector | In vivo validation (Fig. 5, 6) |
| **23** | AAV-PHP.B-U6-sgNeuN#2-BH//CAG-eGFP-KASH-WPRE-hGHpA | Viral Vector | FACS for indel analysis (Fig. 7) |
| **24** | NeuN Forward | Primer | Targeted NGS for indel analysis (Fig. 7) |
| **25** | NeuN Reverse | Primer | Targeted NGS for indel analysis (Fig. 7) |

**Table S2. A list of all sgRNA sequences and primer sequences used in the study**

| **#** | **Name** | **Type** | **Sequence (Spacer-crRNA Repeats-*tracrRNA*)** |
| --- | --- | --- | --- |
| **1** | sgNeuN#1-**FZ** | sgRNA | GACUCCACCCUUCCGACCCCA**GUUUUAGAGCUAGAAA*UAGCAAGUUAAAAUAAGGCUAGUCCGUUAUCAACUUGAAAAAGUGGCACCGAGUCGGUGCUUUUUU*** |
| **2** | sgNeuN#2- **FZ** | sgRNA | GUUUGGGCUGCUGCUUCUCCG**GUUUUAGAGCUAGAAA*UAGCAAGUUAAAAUAAGGCUAGUCCGUUAUCAACUUGAAAAAGUGGCACCGAGUCGGUGCUUUUUU*** |
| **3** | sgNeuN#3- **FZ** | sgRNA | GUCGGGGUCCCUGAACCGGA**GUUUUAGAGCUAGAAA*UAGCAAGUUAAAAUAAGGCUAGUCCGUUAUCAACUUGAAAAAGUGGCACCGAGUCGGUGCUUUUUU*** |
| **4** | sgLacZ- **FZ** | sgRNA | GUGCGAAUACGCCCACGCGAU**GUUUUAGAGCUAGAAA*UAGCAAGUUAAAAUAAGGCUAGUCCGUUAUCAACUUGAAAAAGUGGCACCGAGUCGGUGCUUUUUU*** |
| **5** | sgNeuN#1-**BH** | sgRNA | GACUCCACCCUUCCGACCCCA**GUUUAAGAGCUAUGCUGGAAACAGCA*UAGCAAGUUUAAAUAAGGCUAGUCCGUUAUCAACUUGAAAAAGUGGCACCGAGUCGGUGCUUUUUU*** |
| **6** | sgNeuN#2-**BH** | sgRNA | GUUUGGGCUGCUGCUUCUCCG**GUUUAAGAGCUAUGCUGGAAACAGCA*UAGCAAGUUUAAAUAAGGCUAGUCCGUUAUCAACUUGAAAAAGUGGCACCGAGUCGGUGCUUUUUU*** |
| **7** | sgNeuN#3-**BH** | sgRNA | GUCGGGGUCCCUGAACCGGA**GUUUAAGAGCUAUGCUGGAAACAGCA*UAGCAAGUUUAAAUAAGGCUAGUCCGUUAUCAACUUGAAAAAGUGGCACCGAGUCGGUGCUUUUUU*** |
| **8** | sgLacZ-**BH** | sgRNA | GUGCGAAUACGCCCACGCGAU**GUUUAAGAGCUAUGCUGGAAACAGCA*UAGCAAGUUUAAAUAAGGCUAGUCCGUUAUCAACUUGAAAAAGUGGCACCGAGUCGGUGCUUUUUU*** |
|  |  |  |  |
| **#** | **Name** | **Type** | **Sequence** (**Illumine Adaptor**- Primer) |
| **9** | NeuN Forward | Primer | **TCGTCGGCAGCGTCAGATGTGTATAAGAGACAG**CTAAGGAATCTCGGCTGGAG |
| **10** | NeuN Reverse | Primer | **GTCTCGTGGGCTCGGAGATGTGTATAAGAGACAG**AGTCTGCAGGACTACCTTACAACT |

**Table S3. All equations used to analyze immunohistochemistry slides.** (1) Equation was used to determine the percentage of neuronal cells stained positive for NeuN among all of the cells stained positive for DAPI. (2) Equation was used to determine AAV transduction in all cells. (3) Equation was used to determine the efficiency of CRISPR-mediated disruption of the NeuN gene in neurons stained positive for NeuN.

| (1) | $\% \mathrm{NeuN}^{+}\mathrm{DAPI}^{+} =\frac{\mathrm{DAPI}^{+}\mathrm{NeuN}^{+}}{\mathrm{DAPI}^{+}} \times100\%$ |
| --- | --- |
| (2) | $\% \mathrm{NeuN}^{+}\mathrm{mCherry}^{+} =\frac{\mathrm{NeuN}^{+}\mathrm{mCherry}^{+}}{\mathrm{NeuN}^{+}} \times100\%$ |
| (3) | $\% CRISPR Efficiency=\left( \frac{\mathrm{Control} \left( \%{\mathrm{NeuN}^{+}\mathrm{DAPI}}^{+} \right)-sgNeuN\#2\_BH \left( {{\%NeuN}^{+}\mathrm{DAPI}}^{+} \right)}{\mathrm{Control} \left( {\%\mathrm{NeuN}^{+}\mathrm{DAPI}}^{+} \right)} \right)\times100\%$ |
